# P-724. Benzathine Penicillin G Shortage and Effects on Syphilis Patient Outcomes

**DOI:** 10.1093/ofid/ofaf695.935

**Published:** 2026-01-11

**Authors:** Isa Faghihi, Helen L King, Yoomi Kwon

**Affiliations:** UT Southwestern Medical Center, Dallas, TX; University of Texas Southwestern, Dallas, Texas; Parkland Hospital, Dallas, Texas

## Abstract

**Background:**

A critical benzathine penicillin G shortage in April 2023 led the Centers for Disease Control and Prevention (CDC) to recommend reservation of benzathine penicillin G doses for pregnant individuals and neonates with syphilis. At Parkland Health, oral doxycycline was the recommended treatment for syphilis infections in non-pregnant adults. The impact of doxycycline treatment of syphilis on a population level is unclear.

**Table 1.**
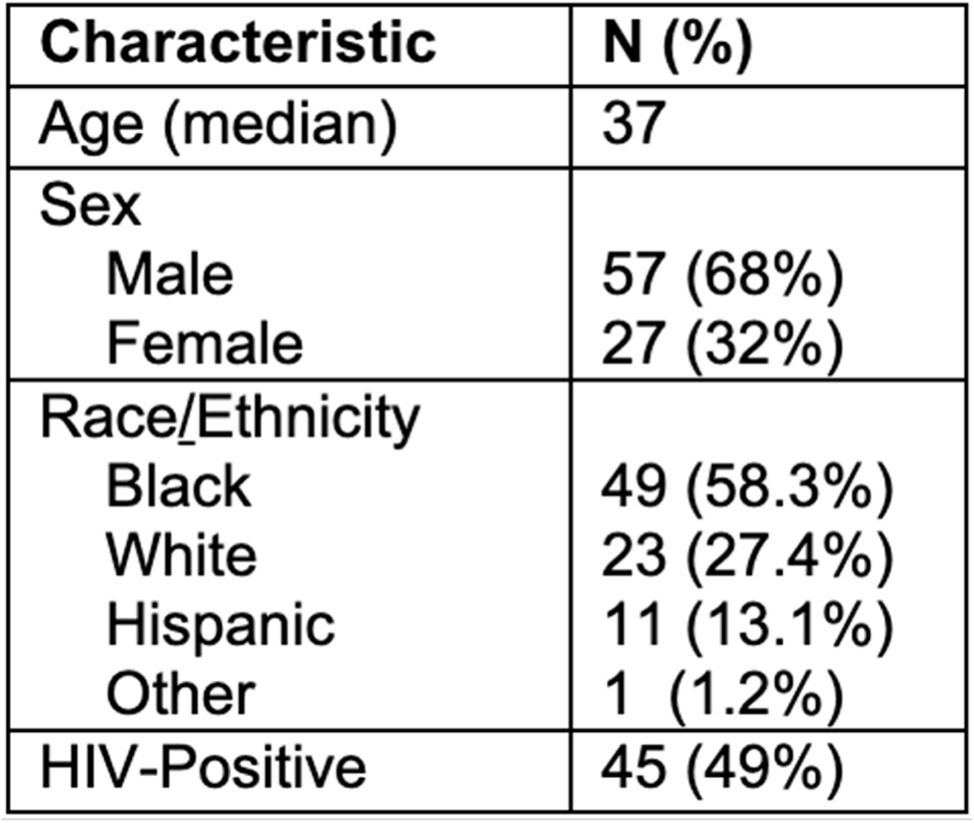
Demographic Characteristics of Individuals Prescribed Doxycycline for Syphilis (n=84)

**Figure:**
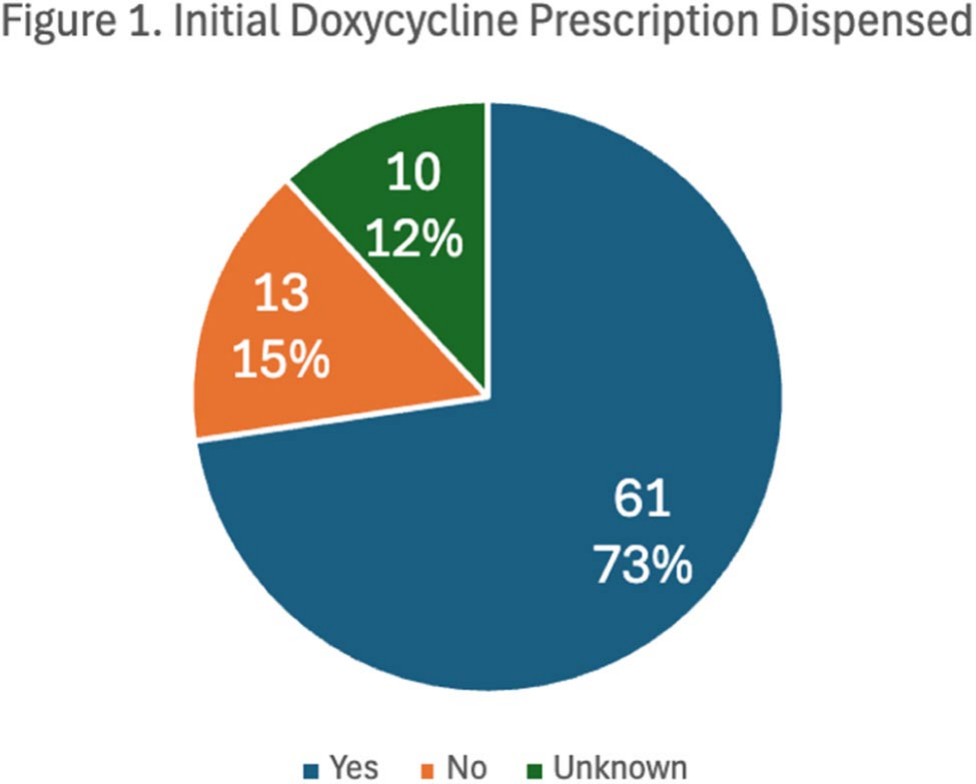

**Methods:**

We performed a retrospective chart review of all patients at Parkland Health who received doxycycline for syphilis from April 2023 to July 2023, including demographic characteristics, HIV status and doxycycline prescription dispensation. We also described treatment completion rates and sexual health outreach.

**Figure:**
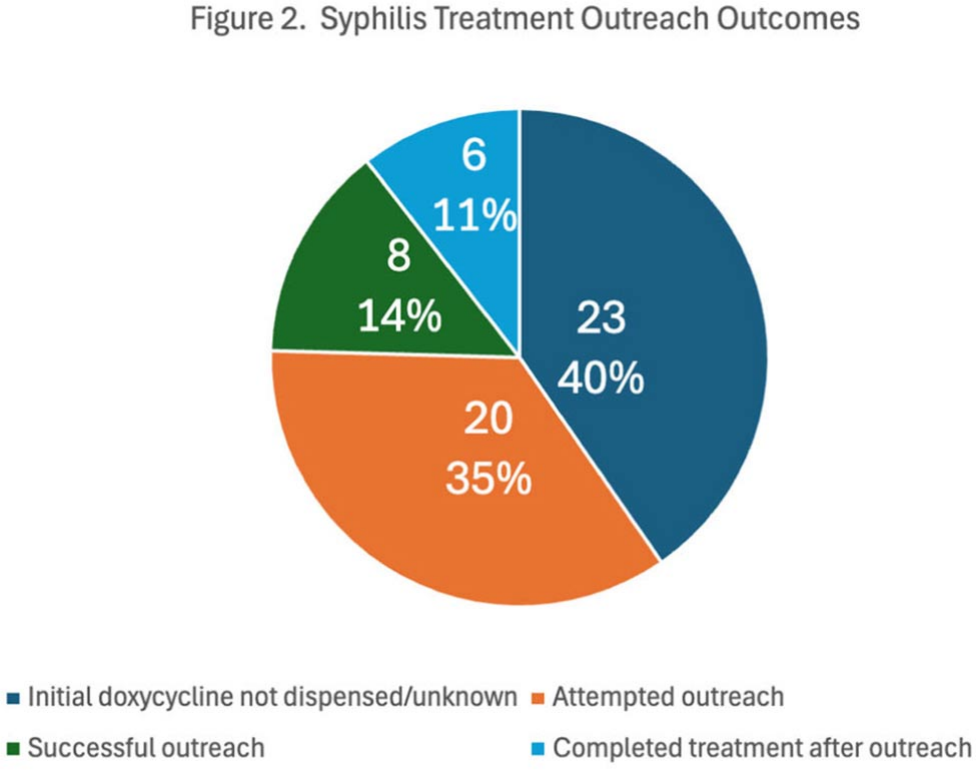

**Results:**

During the study period, 84 individuals were prescribed doxycycline for a syphilis diagnosis. Of those prescribed doxycycline, 61/84 (73%) of the prescriptions were confirmed dispensed, 10/84 (12%) had their prescription sent externally, and 13/84 (15%) were not dispensed. Of those without confirmation of doxycycline dispensation, the sexual health outreach team contacted 20 individuals who had not completed treatment, and an additional 6 were treated after outreach.

**Conclusion:**

With routine use of doxycycline for syphilis during a benzathine penicillin G shortage, only 73% of patients were confirmed to have doxycycline dispensed. Sexual health outreach led to improved completion of treatment of syphilis. Further epidemiologic investigation is needed to assess whether the shortage impacted syphilis transmission and incidence rates.

**Disclosures:**

Helen L. King, MD, Gilead Sciences: Grant/Research Support

